# Snake venom toxin from *vipera lebetina turanica* induces apoptosis of colon cancer cells via upregulation of ROS- and JNK-mediated death receptor expression

**DOI:** 10.1186/1471-2407-12-228

**Published:** 2012-06-08

**Authors:** Mi Hee Park, MiRan Jo, Dohee Won, Ho Sueb Song, Sang Bae Han, Min Jong Song, Jin Tae Hong

**Affiliations:** 1 College of Pharmacy and Medical Research Center, Chungbuk National University, 12 Gaeshin-dong, Heungduk-gu, Cheongju, Chungbuk, 361-763, South Korea; 2 College of Oriental Medicine, Kyungwon University, San 65 Bokjeong-dong, Sujeong-gu, Seongnam, Gyeonggii; 3 Department of Obstetrics and Gynecology, Daejeon St. Mary's Hospital, College of Medicine, The Catholic University of Korea; 4 College of Pharmacy and Medical Research Center, Chungbuk National University, 48 Gaeshin-dong, Heungduk-gu, Cheongju, Chungbuk, 361-763, South Korea

**Keywords:** Snake venom toxin, Apoptosis, Death receptor, ROS, JNK

## Abstract

**Background:**

Abundant research suggested that the cancer cells avoid destruction by the immune system through down-regulation or mutation of death receptors. Therefore, it is very important that finding the agents that increase the death receptors of cancer cells. In this study, we demonstrated that the snake venom toxin from *Vipera lebetina turanica* induce the apoptosis of colon cancer cells through reactive oxygen species (ROS) and c-Jun N-terminal kinases (JNK) dependent death receptor (DR4 and DR5) expression.

**Methods:**

We used cell viability assays, DAPI/TUNEL assays, as well as western blot for detection of apoptosis related proteins and DRs to demonstrate that snake venom toxin-induced apoptosis is DR4 and DR5 dependent. We carried out transient siRNA knockdowns of DR4 and DR5 in colon cancer cells.

**Results:**

We showed that snake venom toxin inhibited growth of colon cancer cells through induction of apoptosis. We also showed that the expression of DR4 and DR5 was increased by treatment of snake venom toxin. Moreover, knockdown of DR4 or DR5 reversed the effect of snake venom toxin. Snake venom toxin also induced JNK phosphorylation and ROS generation, however, pretreatment of JNK inhibitor and ROS scavenger reversed the inhibitory effect of snake venom toxin on cancer cell proliferation, and reduced the snake venom toxin-induced upregulation of DR4 and DR5 expression.

**Conclusions:**

Our results indicated that snake venom toxin could inhibit human colon cancer cell growth, and these effects may be related to ROS and JNK mediated activation of death receptor (DR4 and DR5) signals.

## Background

Colorectal cancer is one of the most common fetal cancers, causing the second cancer-related death [1]. Although a number of chemotherapeutic agents such as capecitabine, irinotecan, oxaliplatin, and leucovorin-modulated fluorouracil have improved response rates to chemotherapy in advanced colorectal cancer [2-4], resistance to chemotherapy remains a major problem in the therapy of this cancer and new approaches are urgently required [5]. Moreover, it is reported that most chemotherapeutics have marked cytotoxic effects on normal cells [6,7]. Recently, a body of evidence suggested that down-regulation or mutation of death receptors (DRs) might be a mechanism by which cancer cells avoid destruction by the immune system [8,9]. Breaking such resistance was rendered by some anticancer drugs that enhance death receptor expression and aggregation at the surface of tumor cells, thereby increasing the apoptotic response to death receptor ligands [8,9]. Therefore, it is very important to find agents that increase the death receptors of cancer cells for decrease of resistance.

Apoptosis is the best characterized form of programmed cell death and is an intracellular suicide program possessing morphologic characteristics and biochemical features, including chromatin condensation, nuclear DNA fragmentation, cell shrinkage, membrane blebbing, and the formation of apoptotic bodies [10,11]. It is an important process in maintaining homeostasis which can be triggered by many factors like radiation and chemotherapeutics drugs [12]. To date, two major apoptotic pathways have been described as follows: the intrinsic mitochondrion-initiated pathway and the extrinsic death receptor-mediated pathway [13,14]. In the intrinsic (mitochondrial) pathway, proapoptotic proteins result in a net increase of free cytosolic cytochrome C. Once released, cytochrome c interacts with adenosine triphosphate, apoptosis-activating factor-1 (Apaf-1) and procaspase 9 to form the apoptosome. The apoptosome cleaves and activates caspase 9, which leads to caspases 3, 6, and 7 activation, thus stimulating apoptosis [15,16]. The extrinsic apoptotic pathway originates at membrane death receptors such as DR4 (TRAIL-R1), and DR5 (TRAIL-R2) and Fas (CD95/APO-1) [17-19]. In this extrinsic pathway, binding of tumor necrosis factor (TNF), TNF-related apoptosis-inducing ligand (TRAIL), or Fas ligands to their receptors, in association with adaptor molecules such as Fas-associated death domain (FADD) or TNF receptor-associated death domain, leads to cleavage and activation of initiator caspase 8 and 10, which in turn cleaves and activates executioner caspases 3, 6, and 7 culminating in apoptosis. Recently, the use of death receptor ligands as therapeutic agents has come under scrutiny [17-21].

The death receptors (DRs) are induced through reactive oxygen species (ROS), mitogen activated protein kinases (MAPKs) and p53 dependent pathway [22-25]. It has been reported that DRs are induced through ROS dependent pathways by several chemotherapeutic agents [22-25]. Previous studies demonstrated that the curcumin induced renal cancer cell apoptosis by induction of DR5 accompanied with the generation of ROS and sensitized TRAIL induced apoptosis. However this apoptotic effect and DR5 upregulation were blocked by treatment of N-acetylcysteine (NAC), a ROS scavenger [22]. Other groups also showed that baicalein and ursolic acid (UA) enhanced ROS-mediated DR4 or/and DR5 expression in colon cancer cells, and thereby enhanced TRAIL-induced apoptosis which was reversed by NAC [23,24]. Several reports demonstrated that MAPKs, including extracellular signal-regulated kinases (ERK)1/2, p38 MAPK, and Jun N-terminal kinase (JNK) also have been shown to mediate up-regulation of DRs [24,25]. LY303511 upregulated DR4 and DR5 by activation of JNK and ERK pathways and enhanced TRAIL induced apoptosis in neuroblastoma cells, and the induction of DRs and TRAIL induced apoptosis were reduced by treatment of JNK and ERK inhibitors [25]. It was also reported that the bisindolylmaleimide induced DR5 expression by JNK and p38 pathways in astrocytoma cells [26].

Many researchers have believed that natural snake venom toxins are useful biological resource, containing several pharmacologically active components that could be of potential therapeutic value [27]. Recently, a lot of effort has been taken to develop snake venom toxin into therapeutics such as anti-hypertensive, anti-coagulant and anti-stroke drugs [28]. Particularly snake venom toxin from *Vipera lebetina turanica* was previously demonstrated as a possible chemotherapeutic against for growth of human prostate cancer cell and neuroblastoma cell through induction of apoptosis via modulating the expression of apoptosis regulatory proteins and ROS dependent mechanisms [27,29]. However, the apoptotic effect of snake venom toxin on colon cancer cells through induction of DR expression has not been studied yet. In this study, we evaluated effects of snake venom toxin obtained from *Vipera lebetina turanica* on colon cancer cells. In particular, we determine the capacity of the venom toxin to suppress colon cancer cell growth by enhancing expression of death receptors through ROS and JNK pathway.

## Methods

### Materials

Snake venom toxin from *Vipera lebetina turanica* was purchased from Sigma (St. Louis, MO). N-acetycysteine and SP600125 were purchased from Sigma. Soluble Recombinant human Apo2L/TRAIL was purchased from Peprotech (Rocky Hill, NJ). Small interfering (si) RNA species for death receptor (DR4 and DR5) and non-targeting control siRNA were purchased from Bioneer (Daejeon, Korea), and death receptor 4 (DR4) was purchased from Santa Cruz Biotechnology Inc. (Santa Cruz, CA, USA)

### Cell culture and regents

HCT116, HT-29 colon cancer cells and CCD18 Co normal colon cell were obtained from the American Type Culture Collection (Manassas, VA). Cells were grown at 37°C in 5% CO_2_ humidified air in RPMI 1640 medium supplemented with 10% fetal bovine serum (FBS), 100 U/ml penicillin, and 100 μg/ml streptomycin. RPMI1640, penicillin, streptomycin and FBS were purchased from Gibco Life Technologies (Grand Island, NY).

### Cell viability

To determine viable cell numbers, the HCT116, HT-29 colon cancer cells and CCD18 Co normal colon cells were seeded onto 24-well plates (5 × 10^4^ cells/well). The cells were trypsinized, pelleted by centrifugation for 5 min at 1500 rpm, resuspended in 10 ml of phosphate-buffered saline (PBS), and 0.1 ml of 0.2% trypan blue was added to the cell suspension in each solution (0.9 ml each). Subsequently, a drop of suspension was placed in a Neubauer chamber, and the living cancer cells were counted. Cells that showed signs of trypan blue uptake were considered to be dead, whereas those that excluded trypan blue were considered to be viable. Each assay was carried out in triplicate.

### Apoptosis evaluation

Detection of apoptosis was done as described elsewhere [27]. In short, cells were cultured on 8-chamber slides. The cells were washed twice with PBS and fixed by incubation in 4% paraformaldehyde in PBS for 1 h at room temperature. TdT-mediated dUTP nick and labeling (TUNEL) assays were performed by using the in situ Cell Death Detection Kit (Roche Diagonostics GmbH, Mannheim, Germany) according to manufacture’s instructions. Total number of cells in a given area was determined by using DAPI staining. The apoptotic index was determined as the number of TUNEL-positive stained cells divided by the total cell number counted x100.

### Western blotting

Western blot analysis was performed as described previously [27]. To prepare the cytosolic extract, the cells were harvested and suspended in an ice-cold solution containing 20 mM HEPES (pH 7.5), 1.5 mM MgCl2, 10 mM KCl, 1 mM EDTA, 1 mM EGTA, 1 mM DTT, 0.1 mM phenylmethylsulfonyl fluoride, 10 μg/ml leupeptin, 10 μg/ml aprotinin, 10 μg/ml pepstatin, and 250 mM sucrose. The cells were disrupted using a Dounce homogenizer. The samples were centrifuged at 1,500 g for 5 min at 4°C to remove nuclei and intact cells. The supernatant was centrifuged at 105,000 g for 30 min at 4°C. The resulting supernatant was used as the soluble cytosolic fraction. The membranes were immunoblotted with the following primary antibodies: mouse monoclonal antibodies directed against cleaved caspase-8 (1:1000 dilutions; Cell Signaling Technology, Beverly, MA) cytochrome-C, p53 and bax (1:500 dilutions; Santa Cruz Biotechnology Inc. CA, USA.), and rabbit polyclonal antibodies directed against ERK, phospho-ERK and JNK (1:500 dilutions; Santa Cruz Biotechnology Inc. CA, USA.), and cleaved caspase-3, -9 and phospho-JNK (1:1000 dilutions; Cell Signaling Technology, Beverly, MA). The blot was then incubated with the corresponding anti-mouse/rabbit immunoglobulin G-horseradish peroxidase-conjugated secondary antibody (Santa Cruz Biotechnology Inc. CA, USA). Immunoreactive proteins were detected with the Enhanced Chemiluminescence Western blotting detection system (Amersham Pharmacia Biotech, Inc., Buckinghamshire, UK). The relative density of the protein bands was scanned by densitometry using MyImage (Seoulin Bioscience Inc., Seoul, Korea) and quantified by Labworks 4.0 software (UVP Inc., Upland, CA, USA).

### Transfection

HCT116, HT-29 colon cancer cells (5 × 10^4^ cells/well) were plated in 24-well plates and transiently transfected with 0.4 μg of the empty vector or the 100 nM of negative siRNA, DR4 or DR5 siRNA per well, using a mixture of plasmid and the WelFect-EX PLUS reagent in OPTI-MEM, according to manufacturer's specification (WelGENE, Seoul, Korea).

### RT–PCR

Total RNA was extracted by RNeasy kit (Qiagen, Valencia, CA, USA). The RT reaction was performed using RNA to cDNA Kit (Applied Biosystems, Foster City, CA, USA). The PCR reaction was performed with cDNA as a template using the primers below after an initial 1-min denaturation at 96°C, followed by the indicated cycles of 96°C for 1 min, 60°C or 63°C for 1 min and 72°C for 1 min. The used PCR primers were 5’-ACCAATGCCACAAAGGAAC-3’ and 5’-CTG CAATTGAAGCACTGGAA-3’ for the human TNF receptor 1, 5’-CTCAGGAGCATG GGGATAAA-3’ and 5’-AGCCAGCCAGTCTGACATCT-3’ for the human TNF receptor-2, 5’-ATGGCGATGGCTGCGTGTCCTG-3’ and 5’-AGCGCCTCCTGGGTCTCGGGGTAG-3’ for the human DR3, 5’-ACTTTGGTTGTTCCGTTGCTG TTG-3’ and 5’-GGCTTTCCATTTGCTGCTCA-3’ for the human DR4, 5’-TGGAACAACGGGGACAGAACG-3’ and 5’-GCAGCGCAAGCAGAAAAGGAG-3’ for the human DR5, 5’-AAGCCGGGGACC AAGGAGACAGACAAC-3’ and 5’-TGCCGGGGCCCTTTTTCAGAG T-3’ for the human DR6 and 5’-CAAAGCCCATTTTTCTTCCA-3’ and 5’-GACAAAGCCACCCCAAGTTA-3’ for human FAS, 5’-CAGCTCTTCCACCTACAG AAG G-3’ and 5’-AAGATTGAACACTGCCCCCAGG-3’ for FasL, 5'-AGACCTGCGTGCTGATCGTG-3' and 5'-TTATTTTGCGGCCCAGAGCC-3' for human TRAIL, 5’-GAAGGTGAAGGxTCGGAGT-3’ and 5’-CTTCTACCACTACCCTAAAG-3’ for glyceraldehyde-3-phosphate dehydrogenase (GAPDH), respectively.

### Measurement of ROS

Generation of ROS was assessed by 2, 7- dichlorofluorescein diacetate (DCFH-DA, Sigma Aldrich, St Louis, MO, USA), an oxidation-sensitive fluorescent probe. Intracellular H_2_O_2_ or low-molecular-weight peroxides can oxidize 2, 7-dichlorofluorescein diacetate to the highly fluorescent compound dichlorofluorescein (DCF). Briefly, cells were plated in 6 well plates (5X10^4^), and subconfluent cells were subsequently treated with snake venom toxin (0.1-1 μg/ml) for 30 min. After the cells were trypsinized, the 1x10^4^ cells were plated in black 96 well plate and incubated with 10 μM DCFH-DA at 37°C for 4 h. The fluorescence intensity of DCF was measured in a microplate-reader at an excitation wavelength of 485 nm and an emission wavelength of 538 nm.

### Statistical analysis

The data were analyzed using the GraphPad Prism 4 ver. 4.03 software (GraphPad Software, La Jolla, CA, USA). Data are presented as mean ± SD. The differences in all data were assessed by one-way analysis of variance (ANOVA). When the *P* value in the ANOVA test indicated statistical significance, the differences were assessed by the Dunnett’s test. A value of *p* < 0.05 was considered to be statistically significant.

## Results

### Effect of snake venom toxin on the growth of human colon cancer cells

**Figure 1:**
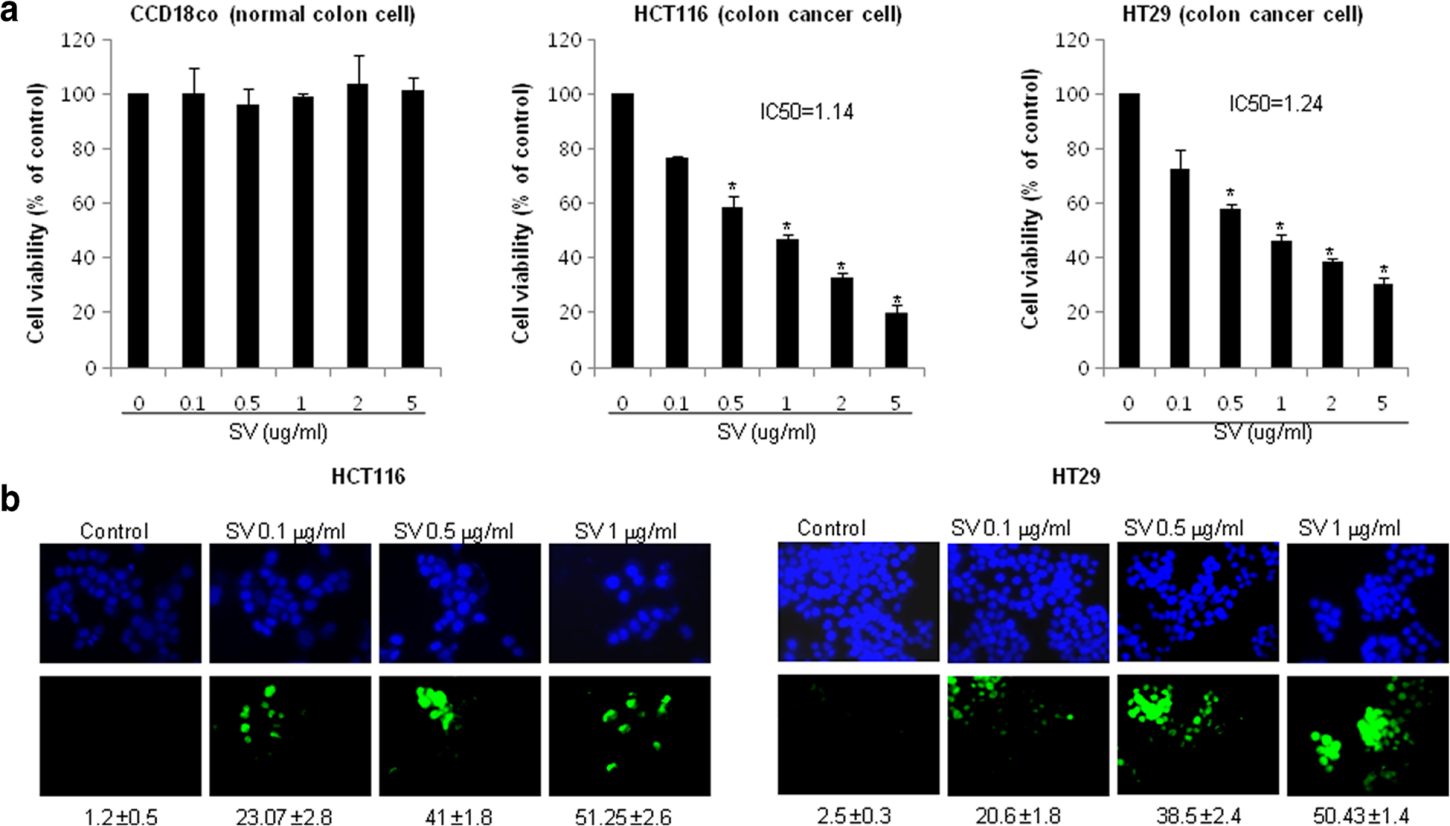
**Effect of snake venom toxin on viability of human colon cancer cells.** HCT116 cells and HT-29 cells were inoculated into 24-well plates (5 × 10^4^ cells/well) and thereafter treated with snake venom toxin (0.1, 0.5, 1 μg/ml) at 37°C for 24 h. **a,** Cell viability of HCT 116 cell, HT-29 cell and CCD18Co cells was determined by direct counting viable cells in Neubauer chamber. The results were expressed as a percentage of viable cells. **b,** Analysis of apoptosis by TUNEL assay. The colon cancer cells (HCT116 and HT-29) were treated with snake venom toxin (0.1-1 μg/ml) for 24 h, and then labeled with TUNEL solution. Total number of cells in a given area was determined by using DAPI nuclear staining (fluorescent microscope). The apoptotic index was determined as the DAPI-stained TUNEL-positive cell number/total DAPI stained cell number (magnification, 200x). *Columns,* means of three experiments, with triplicates of each experiment; *bars,* SD. *, *p* <0.05, significantly different from snake venom toxin-untreated control cells.

To evaluate an effect of the snake venom toxin from *Vipera lebetina turanica* on the growth of colon cancer cells, we analyzed the cell viability by direct counting viable cells in Neubauer chamber. Snake venom toxin (0.1-1 μg/ml) inhibited HCT116 and HT-29 colon cancer cell viability dose dependently. The IC_50_ values of snake venom toxin in HCT116 and HT-29 is 1.14 μg/ml and 1.24 μg/ml, respectively. However, there are no remarkable changes in CCD18 Co normal colon cell viability (Figure 1A). To determine if the inhibition of cell viability by snake venom toxin was due to the induction of apoptosis, we evaluated the changes in the chromatin morphology of cells by using DAPI staining followed by TUNEL staining assays, and then the double labeled cells were analyzed by fluorescence microscope. The cells were treated with various concentrations of snake venom toxin (0.1, 0.5 and 1 μg/ml) for 24 h. DAPI-stained TUNEL-positive cells were concentration-dependently increased and highest concentration of snake venom toxin (1 μg/ml) caused most of cells TUNEL-positive, and the apoptosis rates were 51.25 ± 2.6% in HCT116 cells and 50.43 ± 1.4% in HT-29 cells (Figure 1B). These results demonstrated that snake venom toxin treatment strongly induced apoptosis in colon cancer cells.

### Effect of snake venom toxin on the ROS generation in human colon cancer cells

**Figure 2:**
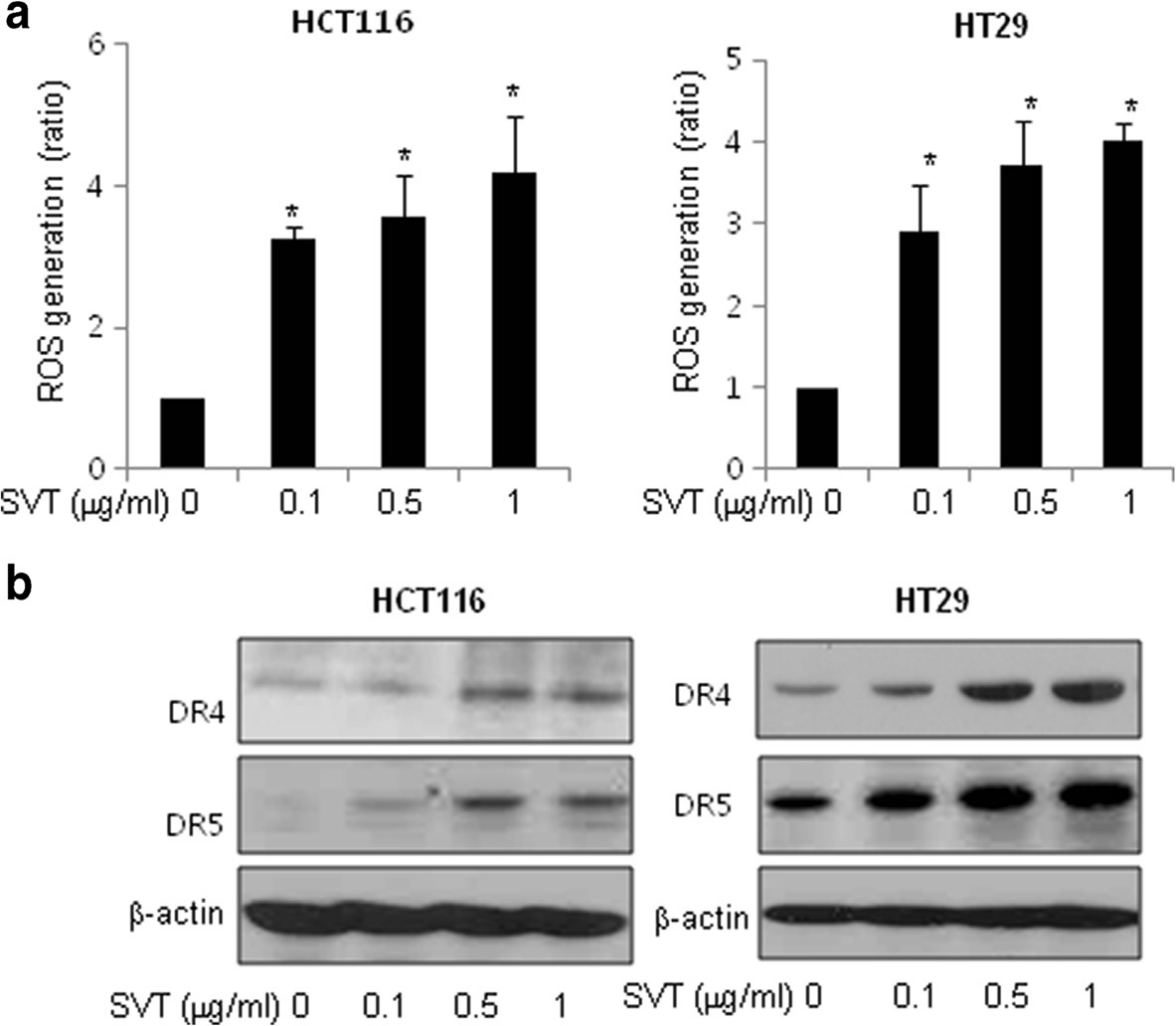
**Effect of snake venom toxin on ROS generation and the expression of death receptors in human colon cancer cells. a,** Effect of snake venom toxin on ROS generation by treatment of snake venom toxin in colon cancer cells. After treatment of snake venom toxin for 30 min, the cells were incubated with 10 μM DCF-DA at 37°C for 4 h, and then washed twice with PBS. The fluorescence intensity of DCF was measured in a microplate-reader at an excitation wavelength of 485 nm and an emission wavelength of 538 nm. **b**, Two colon cancer cells, HCT116 cells and HT-29 cells were treated with snake venom toxin (0.1, 0.5, 1 μg/ml) at 37°C for 24 h, and equal amounts of total proteins (50 μg/lane) were subjected to 12% SDS-PAGE. Expression of DR4, DR5 and β-actin was detected by Western blotting using specific antibodies. β-actin protein was used an internal control. Each band is representative for three experiments. *Columns,* means of three experiments, with triplicates of each experiment; *bars,* SD. *, *p* <0.05, significantly different from non treated control group.

Several chemotherapeutic agents induce apoptosis by increase of ROS [30,31]. We investigated whether snake venom toxin also induced ROS in colon cancer cell lines, since we had found that ROS is implicated in the snake venom toxin-induced neuroblastoma cell death [29]. Thus, we determined the role of ROS in mediating SVT-induced apoptosis of HCT116 and HT-29 cells by measuring ROS levels after treatment of varying concentrations of snake venom toxin (0.1, 0.5 and 1 μg/ml) for 30 min. As shown in Figure 2A, snake venom toxin increased ROS levels in a dose-dependent manner in both HCT116 and HT-29 cells.

### Effect of snake venom toxin on the expression of death receptors in human colon cancer cells

Several studies demonstrated that the ROS generation is involved in DR4 and DR5 upregulation by treatment of chemotherapeutic agents such as curcumin, baicalein and ursolic acid [22-24]. We investigated the possible involvement of ROS in the expression of death receptors after treatment of snake venom toxin. We evaluated changes in expression of several death receptors and their ligands in HCT116 and HT-29 colon cancer cells using RT-PCR. Consistent with the increase of apoptosis, the expressions of DR4 and DR5 was significantly increased by treatment of snake venom toxin in a dose-dependent manner in HCT116 and HT-29 cells. But expression of other death receptors such as TNF-R1, TNF-R2, DR3, DR6 and Fas and death receptor ligands such as FasL and TRAIL was not changed by treatment of snake venom toxin (Additional file 1: Figure 1). The increased expression of DR4 and DR5 was also confirmed by western blotting (Figure 2B). Taken together, these results indicated that snake venom toxin induced apoptosis by up-regulation of DR4 and DR5 in colon cancer cells.

### Effect of snake venom toxin on the expression of caspase-3, 8, 9 and bax in human colon cancer cells

**Figure 3:**
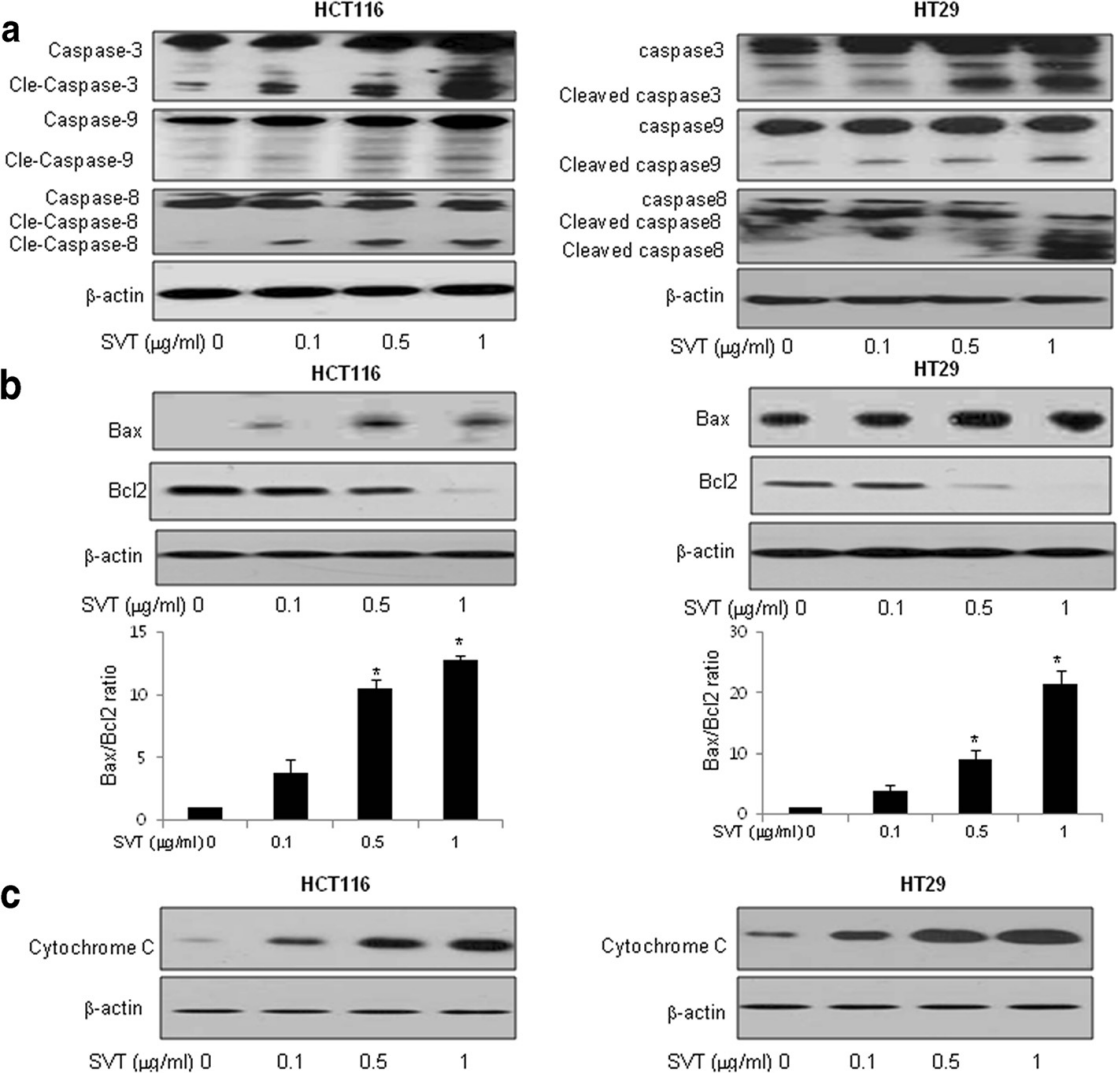
**Effect of snake venom toxin on the expression of apoptosis regulatory proteins in human colon cancer cells. a**, HCT116 cells and HT-29 cells were treated with different concentrations (0.1, 0.5, 1 μg/ml) of snake venom toxin at 37°C for 24 h. Equal amounts of total proteins (50 μg/lane) were subjected to 12% SDS-PAGE. Expression of cleaved caspase-3, cleaved caspase-8 and cleaved caspase-9 was detected by Western blotting using specific antibodies. **b**, HCT116 cells and HT-29 cells were treated with different concentrations (0.1, 0.5, 1 μg/ml) of snake venom toxin at 37°C for 24 h. Equal amounts of total proteins (50 μg/lane) were subjected to 12% SDS-PAGE. Expression of Bax, Bcl2 and β-actin was detected by Western blotting using specific antibodies. *Columns,* means of three experiments, with triplicates of each experiment; *bars,* SD. *, *p* <0.05, significantly different from non treated control group. **c**, HCT116 cells and HT-29 cells were treated with different concentrations (0.1, 0.5, 1 μg/ml) of snake venom toxin at 37°C for 24 h. And cytosol extract was prepared as described in methods. Equal amounts of total proteins (50 μg/lane) were subjected to 12% SDS-PAGE. Expression of cytochrome C and β-actin was detected by Western blotting using specific antibodies. β-actin protein was used an internal control. Each band is representative for three experiments.

To elucidate the relationship between apoptosis and the expression of apoptosis regulatory protein by snake venom toxin, expression of caspase-3, 8, 9, Bax and cytochrome C was investigated since these are DR related down signal cell death proteins. Cells were treated with snake venom toxin (0.1-1 μg/ml), and whole-cell extract was subjected to Western blotting. An increase in the cleavage of caspase-3 (including cleaved caspase-3), caspase-8 (including cleaved caspase-8) and caspase-9 (including cleaved caspase-9) was observed (Figure 3A), Bax/Bcl2 ration was significantly increased (Figure 3B), and the cytochrome C was increased in cytosol extract (Figure 3C) in HCT116 and HT-29 colon cancer cells.

### Effect of knockdown of DR4 and DR5 in snake venom toxin-induced apoptosis

**Figure 4:**
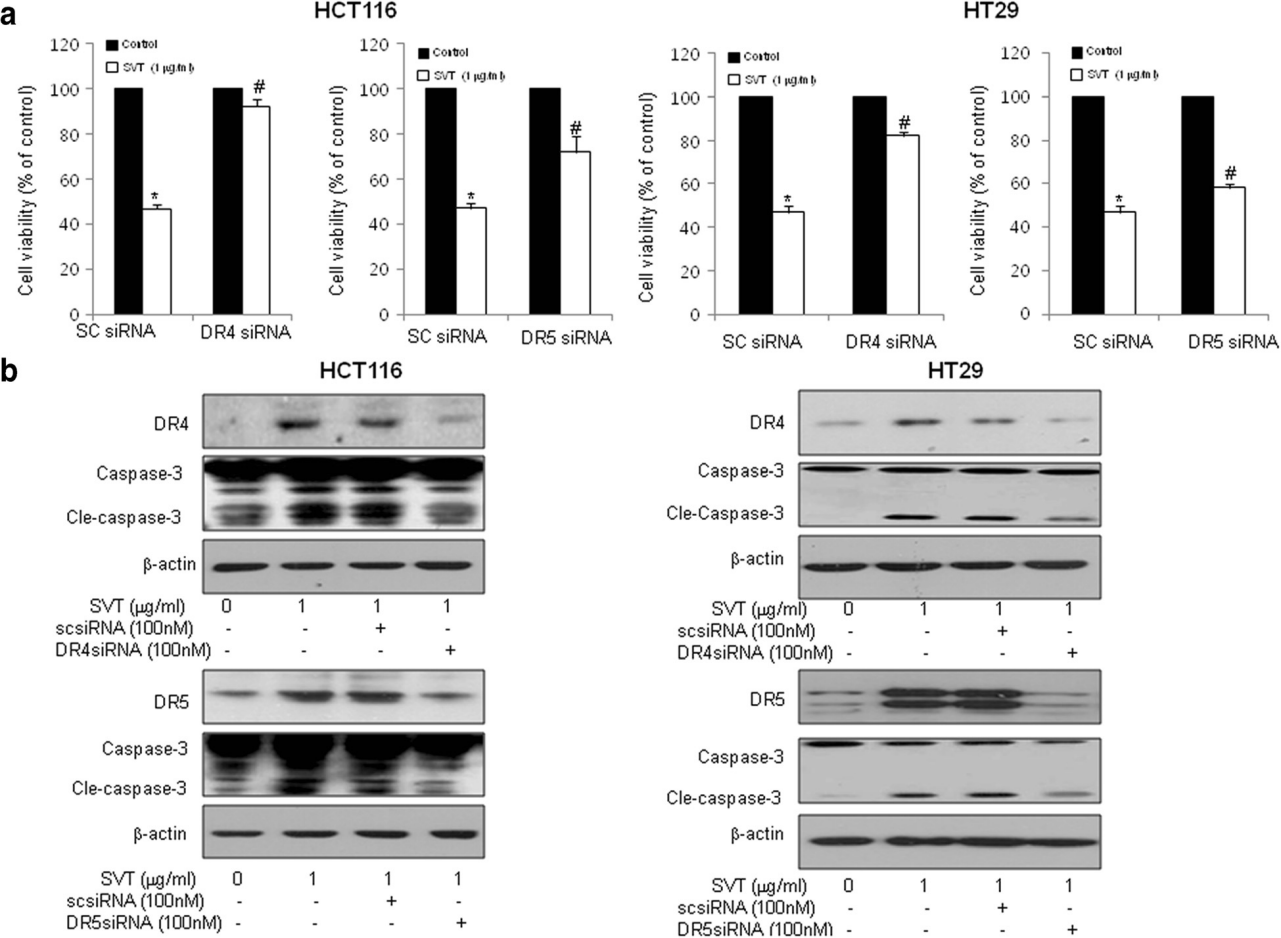
**Effects of DR4 or DR5 knockdown on snake venom toxin induced cell viability inhibition and caspase-3 activation. a,** HCT116 cells and HT-29 cells were transfected with non targeting control siRNA or DR4 or DR5 siRNA (100 nM) as described in Methods for 24 h. Then, implemented snake venom toxin was treated (1 μg/ml) for another 24 h. Thereafter, cell viability was measured by direct counting after trypan blue staining. **b,** Equal amounts of total proteins (50 μg/lane) were subjected to 12% SDS-PAGE. Expression of DR4, DR5, cleaved caspase-3 and β-actin was detected by Western blotting using specific antibodies. β-actin protein was used an internal control. Each band is representative for three experiments. *Columns,* means of three experiments, with triplicates of each experiment; *bars,* SD. *, *p* <0.05, significantly different from non treated control group. #, p <0.01 significantly different from sc siRNA -treated group.

We next investigated the effect of knockdown of DR4 and DR5 on the snake venom toxin induced colon cancer cell viability inhibition using DR4 or DR5 specific siRNA to confirm that the DR4 and DR5 play a critical role on cell death. Figure 4A revealed that the effect of snake venom toxin-induced cell death was effectively abolished in cells transfected with either DR4 or DR5 siRNA (100 nM) in both HCT116 and HT-29 cells. Interestingly, knockdown of DR4 more significantly reversed the growth inhibitory effect of snake venom toxin in HCT116 and HT-29 cells. We also showed that the caspase-3 activation was inhibited by treatment of DR4 or DR5 siRNA accompanied with downregulation of DR4 or DR5 proteins (Figure 4B). These results indicate that DR4 and DR5 play a major role in apoptotic colon cancer cell death by snake venom toxin.

### Involvement of JNK pathway and ROS in the induction of death receptors and apoptosis by snake venom toxin

**Figure 5:**
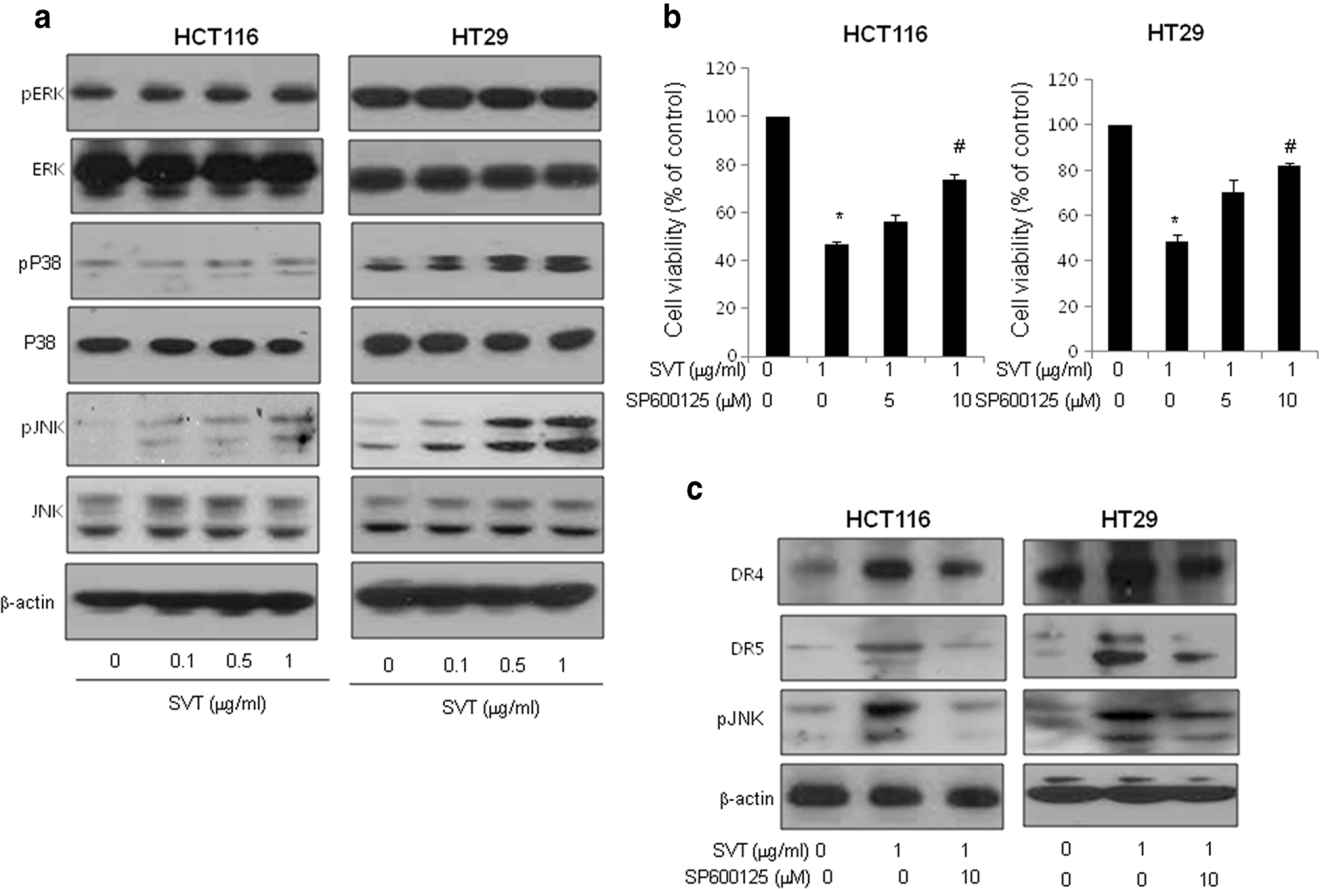
**Effect of JNK pathway on the upregulation of DR4 or DR5, and cell death by snake venom toxin. a**, Effect of snake venom toxin on the expression of MAPK proteins in colon cancer cells. HCT116 cells and HT-29 cells were treated with snake venom toxin for 24 h and whole cell extracts were analyzed by western blotting using the relevant antibodies. **b**, Effect of SP600125 on the cell viability in snake venom toxin treated cancer cells. Cells were pretreated with SP600125 (0, 5, 10 μM) for 1 h and then treated with snake venom toxin for 24 h. The results were expressed as a percentage of viable cells. **c**, Effect of JNK inhibitor (SP600125) on the expression of death receptors. Cells were pretreated with SP600125 (10 μM) for 1 h, and then cells were treated with snake venom toxin for 24 h, and whole cell extracts were analyzed by Western blotting using DR4, DR5, p-JNK and β-actin antibodies. Each band is representative for three experiments. *Columns,* means of three experiments, with triplicates of each experiment; *bars,* SD. *, *p* <0.05, significantly different from non treated control group. #, p <0.01 significantly different from SVT-treated group.

**Figure 6:**
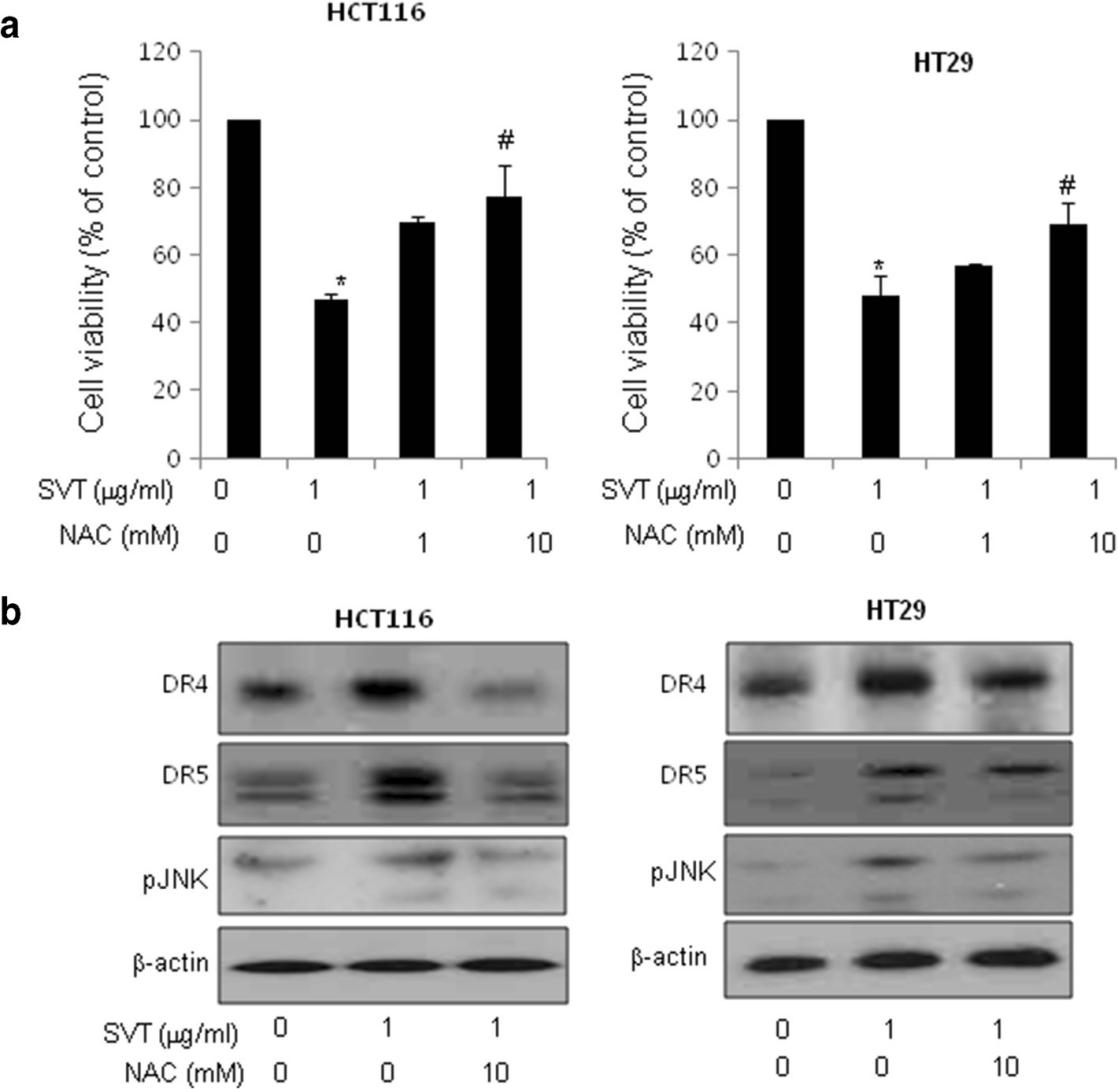
**Effect of ROS on upregulation of DR4 or DR5 through JNK activation by snake venom toxin. a**, Effect of antioxidant (NAC) on the cell viabilty induced by snake venom toxin. Cells were pretreated with various concentratins of NAC (0, 1, 10 mM) for 1 h and then treated with 1 μg/ml of snake venom toxin for 30 min, and whole cell extracts were analyzed by western blotting using the relevant antibodies. **b**, Effect of NAC on the expression of death receptors and JNK phosphorylation. Cells were pretreated with NAC for 1 h, and then cells were treated with snake venom toxin for 30 min, and whole cell extracts were analyzed by Western blotting using DR4, DR5, p-JNK and β-actin antibodies. Each band is representative for three experiments. *Columns,* means of three experiments, with triplicates of each experiment; *bars,* SD. *, *p* <0.05, significantly different from non treated control group. #, p <0.01 significantly different from SVT-treated group.

We found that the JNK was activated by treatment of snake venom toxin, but not ERK and p38 in HCT116 and HT-29 colon cancer cells (Figure 5A). To further investigate whether JNK plays a critical role in snake venom toxin-induced up-regulation of DR4 and DR5, we pretreated the colon cancer cells with SP600125, a JNK inhibitor (5 and 10 μM) for 1 h, and then these cells treated with snake venom toxin (1 μg/ml) for 24 h to assess cell viability and DR4 and DR5 expression. As a result, JNK inhibitor abolished snake venom toxin-induced inhibition of cell viability (Figure 5B) and suppressed the snake venom toxin-induced up-regulation of DR4 and DR5 (Figure 5C), suggesting that JNK pathway may be involved in snake venom toxin-induced apoptosis and upregulation of DRs. Because we already showed that snake venom toxin (0.1-1 μg/ml) induced ROS in a dose-dependent manner in HCT116 and HT-29 cells in Figure 2A, we further investigated whether ROS plays a role in snake venom toxin-induced up-regulation of DR4 and DR5. We pretreated with NAC, an antioxidant (1 and 10 mM) for 1 h in HCT116 and HT-29 cells, and then treated with snake venom toxin (1 μg/ml) for 30 min to assess cell viability and DR4 and DR5 expression. It was found that NAC abolished snake venom toxin-induced inhibition of cell viability (Figure 6A) and suppressed the snake venom toxin-induced up-regulation of DR4 and DR5, and JNK phosphorylation (Figure 6B), suggesting that ROS is also involved in snake venom toxin-induced apoptosis and upregulation of DRs, and activation of JNK. Taken together, these results indicated that the JNK and ROS pathway are critical in induction of DR4 and DR5 expression, and DR4 and DR5 mediated apoptosis by snake venom toxin in colon cancer cells.

## Discussion

We showed that snake venom toxin inhibited HCT116 and HT-29 colon cancer cell growth through apoptosis. Our study also showed that this effect was associated with the JNK and ROS-mediated increased expression of the DR4 and DR5. The TRAIL receptors, DR4 and DR5 are also expressed in colon carcinomas and their expressions are increased as tumor cells acquire malignant potential [32-35]. Colon cancer cells and tumor are relatively sensitive to TRAIL-mediated apoptosis, but normal colonic epithelium are resistant to TRAIL-mediated apoptosis [36-38]. Due to its selective ability for killing of tumor cells with little side effects on normal cells, the activators of TRAIL pathway have emerged as attractive candidates for cancer therapy. It has been shown that TRAIL-induced apoptosis can be enhanced by chemotherapy in several in vitro and xenograft models of cancer, an effect reported to be mediated through increased DR4 and DR5 expression [36-38]. For example, Garcinol derived from dried rind of the fruit *Garcinia indica* has a synergistic anticancer effect with TRAIL by up-regulate the DR4 and DR5 in human colon cancer cells [36]. Celastrol, a triterpenoid isolated from the traditional Chinese medicine enhances TRAIL-induced apoptosis through the upregulation of DRs in colon cancer cells [37]. Diosgenin, a steroid saponin present in fenugreek (*Trigonella foenum graecum*) induced apoptosis in colon cancer cells and sensitized colon cancer cells to TRAIL by induction of DR5 [38].

Recent studies indicate that DR levels can be enhanced by endogenous induction or exogenous overexpression. Several genotoxic and nongenotoxic agents can induce apoptosis by increasing endogenous DRs [39]. On the other hand, exogenously overexpressed DRs, without concomitant up-regulation in its ligand levels, have been shown to be associated with induction of apoptosis [40,41]. In this study, our results demonstrated that SVT-induced apoptosis is coupled with DR4 and DR5. Similar to previous studies, we showed that the snake venom toxin induced DR4 and DR5 in colon cancer cells, however the expression of Fas and other death receptors were not induced. Moreover, we also found that treatment of DR4 or DR5 siRNA reversed snake venom toxin-induced inhibition of cell viability, thus, the inhibitory effect of snake venom toxin could be related with the increase of DR4 and DR5 expression. Caspases play a critical role in apoptosis [42]. Caspase-8 is the most proximal caspase that transmits apoptotic signals originating from the DRs. Activation of caspase- 8 results in activation of downstream caspases such as caspase-3, -6, or −7 and triggering Bax, cytochrome C and caspase-9 apoptosis signal [43]. We showed that the caspase-8 was activated by treatment of snake venom toxin, accompanied with the activation of caspase-3 and −9, expression of Bax and cytosolic release of cytochrome C in a dose dependent manner. Other researchers demonstrated that the Ursodeoxycholic acid (UDCA) induces apoptosis in human gastric cancer cells, and this effect is dominantly mediated by activation of caspase-3, -6 and −8 through increased expression of DR5 [44]. Tocotrienols, a naturally occurring form of vitamin E, also induced apoptosis of breast cancer cells by induced activation of caspase-3 -8 and −9 by upregulation of DR5 [43]. For these reseasons, snake venom toxin may be effective for inducing colon cancer cell death through activation of DR mediated cell death signals.

It has been significantly proposed that the ROS generations are involved in DR4 and DR5 upregulation by chemotherapeutic agents [22-25]. Other previous studies demonstrated that the expression of DR4 and DR5 was induced by several anti-cancer coumpunds shch as curcumin, baicalein and ursolic acid accompanied with the generation of ROS, and these DR4 and DR5 upregulation was blocked by treatment of NAC [21-23]. Consistent with these result, we showed that snake venom toxin induced generation of ROS, and the antioxidant NAC abolished the upregulation of DR4 and DR5 induced by snake venom toxin, and cell growth inhibitory effect by SVT was also reversed by treatment of NAC. Several studies demonstrated that ROS is also significant for the activation of JNK pathway in cancer cell apoptosis. In fact, ROS-dependent activation of JNK is involved in apoptosis, autophage, innate immunity and lifespan limitation [45,46]. Indeed, the activities of ROS and JNK induced by death receptors appear to be linked, both being obligatory participants in the same death-inducing pathway triggered by these receptors [47,48]. It has been demonstrated that several chemotherapeutic agents such as surfactin and celastrol induced apoptosis by induction of ROS through activation of JNK pathway in cancer cells [49,50]. Thus it is also possible that increased ROS by snake venom toxin activates JNK pathway which resulted in upregulation of DR4 and DR5 leading to increase cell death signals. In this study, we showed that the JNK is activated by treatment of snake venom toxin in both HCT116 and HT29 cell lines. Furthermore, JNK inhibitor SP600125 abolished snake venom toxin-induced DR4 and DR5 expression. We also showed that the NAC abolished snake venom toxin-induced JNK phosphorylation accompanied with the activation of DR4 and DR5. These data suggest that activated ROS and consequent activation of JNK could be involved in increased DR4 and DR5 expression. Similar to our results, other groups showed that the tocotrienols induced apoptosis of breast cancer cells by upregulation of DR5 by activation of JNK, p38 MAPK and C/EBP homologous protein (CHOP). Silencing either JNK or p38 MAPK reduced the increase in DR5 and CHOP expression, and blocked tocotrienols-induced apoptosis [43]. It has been also reported that the LY303511 upregulated DR4 and DR5 by activation of JNK in neuroblastoma cells, and the induction of DRs were reduced by treatment of JNK and ERK inhibitors [25]. It was also reported that the bisindolylmaleimide induced the DR5 by activation of JNK and p38 pathways in astrocytoma cell death [26]. And like our studies, other group suggested that melittin, a bee venom toxin compound enhanced TRAIL-induced apoptosis by activating JNK/p38 pathway [51].

Transcriptional regulation of DR4 and DR5 is complex, and multiple potential binding sites of various transcription factors, including p53, are present in the upstream region of DR4 and DR5 [52]. However, we found that the p53 is not induced by snake venom toxin (data not shown). Thus, the induction of DR4 and DR5 by snake venom toxin occurs independent of p53 in colon cancer cells. Instead, our data indicate that snake venom toxin-induced upregulation of DR4 and DR5 could be dependent on the ROS and JNK pathway.

Taken together, our results provide the mechanistic evidence that snake venom toxin treatment results in induction of apoptosis of colon cancer cells through ROS and JNK-mediated upregulation of DR4 and DR5. These results also indicate that snake venom toxin may sensitize colon cancer cells to the TRAIL induced apoptosis. Therefore, our results suggest that the treatment of snake venom toxin could be applicable as an anti-colorectal cancer agent, and/or an adjuvant agent for other chemotherapeutics.

## Conclusions

We demonstrated here that the snake venom toxin from *Vipera lebetina turanica* induced the apoptosis of colon cancer cells through reactive oxygen species (ROS) and c-Jun N-terminal kinases (JNK) dependent death receptor (DR4 and DR5) expression.

## Competing interests

The authors declare that they have no competing interests.

## Authors’ contributions

Mi Hee Park conceived and designed the study, performed experiments, participated in data collection, analyzed the data, and drafted the manuscript. MiRan Jo contributed to the study design, performed experiments, and analyzed data. Dohee Won participated in study design and carried out experiments. Ho Sueb Song participated in study design and manuscript preparation. Sang Bae Han participated in data analysis and manuscript preparation. Min Jong Song participated in study design and manuscript preparation. Jin Tae Hong designed the study, contributed to data collection and analysis, and drafted the manuscript. All authors read and approved the final manuscript.

## Pre-publication history

The pre-publication history for this paper can be accessed here:

http://www.biomedcentral.com/1471-2407/12/228/prepub

## Supplementary Material

Additional file 1 **Figure S1.** Effect of snake venom toxin on the expression of death receptors in human colon cancer cells. HCT116 cells and HT-29 colon cancer cells were treated with snake venom toxin (0.1, 0.5, 1 μg/ml) at 37 °C for 24 h, and total RNA were extracted and examined for expressions of TNF-R1, TNF-R2, DR3, -4, -5, -6, TRAIL, Fas, FasL and GAPDH by RT-PCR. GAPDH was used as an internal control to show equal RNA loading. Each band is representative for three experiments. Click here for file
