# Supplementary figures and images for: Snake venom toxin from vipera lebetina turanica induces apoptosis of colon cancer cells via upregulation of ROS- and JNK-mediated death receptor expression
Source: BMC Cancer. 2012 Jun 8;12:228. doi: 10.1186/1471-2407-12-228 (PMC3584847; doi:10.1186/1471-2407-12-228)

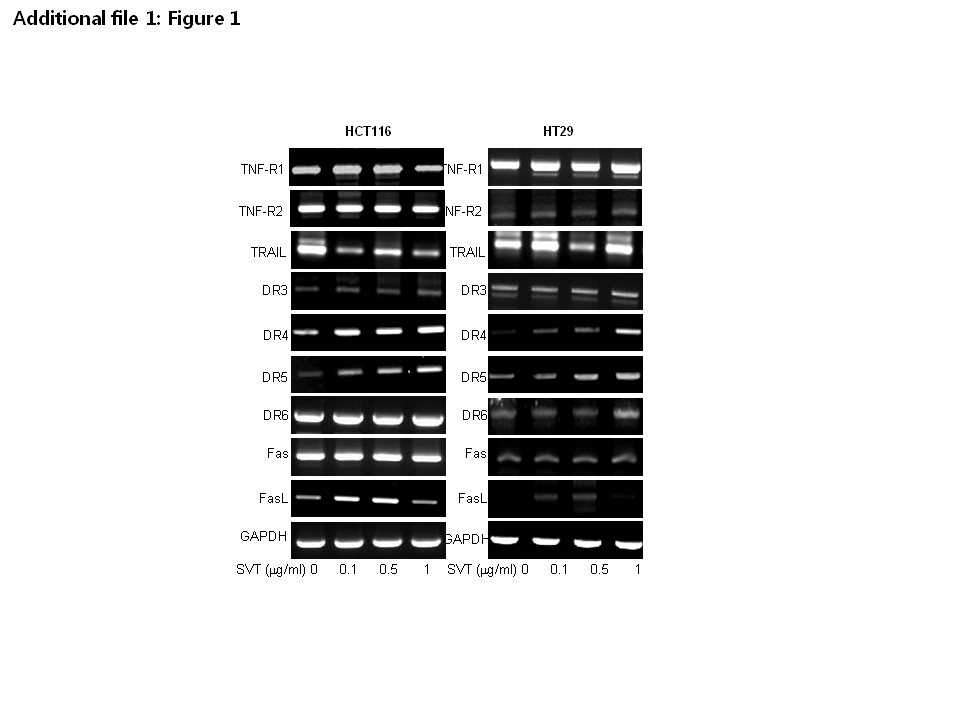

Supplement: Additional file 1 — Figure S1. Effect of snake venom toxin on the expression of death receptors in human colon cancer cells. HCT116 cells and HT-29 colon cancer cells were treated with snake venom toxin (0.1, 0.5, 1 μg/ml) at 37 °C for 24 h, and total RNA were extracted and examined for expressions of TNF-R1, TNF-R2, DR3, -4, -5, -6, TRAIL, Fas, FasL and GAPDH by RT-PCR. GAPDH was used as an internal control to show equal RNA loading. Each band is representative for three experiments. [file 1471-2407-12-228-S1.tiff]
